# Editorial: The Role of Medicinal Plants and Natural Products in Modulating Oxidative Stress and Inflammatory Related Disorders

**DOI:** 10.3389/fphar.2022.957296

**Published:** 2022-06-24

**Authors:** Gabriel A. Agbor, Mario Dell'Agli, Jules-Roger Kuiate, Opeolu Ojo

**Affiliations:** ^1^ Centre for Research on Medicinal Plants and Traditional Medicine, Institute of Medical Research and Medicinal Plants Studies, Yaoundé, Cameroon; ^2^ Laboratory of Pharmacognosy, Department of Pharmacological and Biomolecular, Sciences, Università Degli Studi di Milano, Milan, Italy; ^3^ Department of Biochemistry, Faculty of Sciences, University of Dschang, Dschang, Cameroon; ^4^ Department of Biology, Chemistry and Forensic Science, School of Sciences, Faculty of Science and Technology, University of Wolverhampton, Wolverhampton, United Kingdom

**Keywords:** medicinal plants, natural Products, oxidative Stress, inflammatory disorders, antioxidants, experimental pharmacology, drug discovery

## Introduction

This Research Topic focused on elucidating the role of medicinal plants and natural products in oxidative and inflammatory reactions in different pathological conditions. Although these processes are required in normal physiological functions of the living systems, their exacerbation has been associated with degenerative processes and metabolic diseases. Oxidative stress implicated in the aggravation of neurodegenerative diseases such as Parkinson’s disease, or Alzheimer’s, or others such as AIDS or kidney failure. It is therefore necessary to control this condition and medicinal plants are important sources of antioxidant substances that can help fight against oxidative stress. This is because medicinal plants are a rich source of active compounds known to modulate a variety of pharmacological processes including oxidative stress and inflammation, bringing about wellbeing and stability to living systems. The Research Topic attracted so many researchers to publish original articles or review manuscripts, of which only 20 articles were published (two reviewed and 18 research articles). The editorial highlights the quality of the published articles in this Research Topic.

Osteoarthritis (OA) is the most frequent and disabling inflammatory disease in the elderly. In OA, inflammatory cytokines (IL-1β and TNFα) promote the catabolic activities of cartilage tissues. To counteract OA discomfort, Zhang et al. investigated the importance of xanthohumol (XH), a natural prenylflavonoid originally isolated from hops and beer, in attenuating inflammation. The authors observed that XH inhibited inflammatory responses and ameliorated ECM degradation. This was characterized by low production of NO, PGE2, TNFα, and IL-6. A decreased expression of MMP-3/-13 and ADAMTS-4/-5 and an increased expression of collagen-II and aggrecan were also observed. XH equally activated HO-1 signaling and attenuated IL-1β-induced C/EBPβ promoting the interaction between HO-1 and C/EBPβ, the effect was due to inhibition of the nuclear translocation of C/EBPβ. Pain remains the major symptom for inflammation and provides discomfort to the patient. Henneh et al. showed that hydro-ethanolic extract from *Ziziphus abyssinica* Hochst. Ex A. Rich. (ZAE) root bark as well as isolated compounds (β-amyrin and polpunonic acid) possess analgesic properties. ZAE markedly reduced paw licking responses and writhing in both AAT and FT and attenuated both acute and chronic musculoskeletal pain. The extract also reversed hyperalgesia induced by intraplantar injection of PGE2, bradykinin, TNF-α, and IL-1β. The mechanism of action underlining the analgesic effect was justified through opioidergic, ATP sensitive K+ channels and NO- GMP pathways. Both β-amyrin and polpunonic acid exhibited analgesic activity in the tail suspension test.

Inflammation also plays an important role in neurodegenerative diseases such as Alzheimer and Parkinson. Thus, therapeutic strategies to impair neurodegenerative diseases involve anti-inflammatory drugs. A polypeptide fraction k (ABPPk) of *Achyranthes bidentata* Blume protected neurons and suppressed microglia and astrocyte activation in Parkinson disease mice model. Ge et al. reported the beneficial effect of ABPPk pre-treatment in amyloid β oligomers (AβOs) induced neuroinflammation in Alzheimer disease. This was characterized by the inhibition of pro-inflammatory cytokines mRNA levels in BV2 cells and primary microglia induced by AβOs. In the same way, pre-treatment with ABPPk reduced neurotoxicity of BV2 microglia-conditioned media on primary hippocampal neurons and down-regulated the phosphorylation of IκBα and NF-κB p65 as well as the expression of NLRP3 induced by AβOs (Ge et al.). *In vivo* pre-administration of ABPPk alleviated memory deficits, improved locomotor activity and rescued neuronal degeneration and loss in the hippocampus of AβOs-injected mice. ABPPk can restore the autophagy of microglia damaged by AβOs, thus playing a role in regulating neuroinflammation and alleviating neurotoxicity (Ge et al.). Though being an essential element, when accumulated inside neurons, zinc induces oxidative stress and mitochondrial dysfunction. Chen et al. reported that emodin, a derivative of anthraquinone improved zinc-induced altered expression of phosphorylated ERK1/2 (not total ETK1/2) and synaptic proteins (presynaptic SNAP 25, synaptophysin and postsynaptic PSD95) level in SH-SY5Y cells. Emodin also possessed antioxidant role through inhibition of reactive oxygen species generation; emodin also caused a drop of mitochondrial membrane potential in SH-SY5Y cells, hence exerting a neuroprotective effect. In another study, emodin extracted from *Polygonum multiflorum* Thunb. was shown to possess protective effects against sepsis related intestinal mucosal barrier through its anti-inflammatory and anti-oxidative stress activities Shang et al*.* Emodin increased mRNA and protein expression of Vitamin D Receptor and its downstream molecules *in vitro* and *in vivo* studies. Moreover, emodin inhibited the expression of IL-6, TNF-α and MDA in tissues and serum, and increased the levels of SOD and GSH. Hu et al. observed that Pilose antler peptide (PAP), attenuated the behavior alteration caused by CUMS stimulation, decreased the number of neurons, and restored the dendritic spine density. PAP treatment effectively upregulated the expression of p-AMPK and Sirt1 and suppressed the expression of AcNF-κB, NLRP3, Ac-Caspase-1, GSDMD-N, Cleaved-IL-1β, and Cleaved-IL-18. PAP also selectively inhibited Sirt1 and AMPK thereby compromising its therapeutic effect on depression. Obstructive pulmonary disease (pneumonia) is another chronic inflammatory disorder. Sun et al*.* elucidated the molecular mechanism of action of a Chinese traditional medicine recipe [QingFei Yin (QFY)] against bacterial lung infections. Using two pneumonia models (*in vitro* and *in vivo*), QFY exerted prominent anti-pneumonia effects as it triggered autophagy via downregulation of upstream NLRP3/mTOR signaling pathway events. Dextran sulfate sodium (DSS) is an important compound for the induction of colitis. Yuan et al. isolated a major sesquiterpene lactone (Dehydrocostus lactone (DCL)) from *Aucklandia lappa* DC. that presented therapeutic effects against DSS-induced colitis. DCL improved symptoms related to colitis and colonic barrier injury and inhibited the expression of proinflammatory cytokines and myeloperoxidase in colon tissues. DCL suppressed the phosphorylation and degradation of IκBα and NF-κB nuclear translocation and enhanced the nuclear accumulation of Nrf2 in LPS/IFNγ-treatedRAW264.7 cells. DCL also interacts directly with IKKα/β and Keap1, inhibiting the NF-κB signaling and activating Nrf2 pathway (Yuan et al.). Xu et al. also reported the anti-inflammatory activity of *Alhagi pseudalhagi* (M. Bieb) Desv. Ex B. Keller and Shap. extract (APE) in ulcerative colitis (UC). APE repaired the UC-induced colon mucosa injury, reduced weight loss, attenuated DAI, colon macroscopic damage index, histological inflammation, and downregulated the levels of inflammatory markers in serum and colon tissues. Additionally, APE treatment reduced the levels of TLR4 and phosphorylation of p-NF-κB and p-IK-Kβ. Liu et al. evaluated the effects and mechanisms of separate and combined application of Scutellariae and Coptidis decoction in UC induced in mice. The results revealed that Scutellaria-Coptis relieved colon inflammation in mice exerting an effect on UC. The 16S rRNA sequencing showed that Scutellaria-Coptis exhibited increased microbial diversity and improved intestinal flora composition. Scutellaria-Coptis greatly relieved UC when administered as a combined decoction. Artusa et al. investigated the immunomodulatory properties of coffee extracts from green (GCE) and medium-roasted (RCE) *Coffea canephora* Pierre ex A. Froehner beans in human macrophages. This was characterized in an LPS-induced inflammation model in THP-1-derived human macrophages (TDM). They were able to show a decrease in the number of inflammatory markers (TNF-α, IL-6 and IL-1β) and a concentration-dependent inhibition of the release of interferon-β (IFN-β). A diminished nuclear translocation of p-IRF-3, (the main transcription factor responsible for IFN-β synthesis) was the main molecular mechanism of the inhibition of IFN-β. 5-O-caffeoylquinic acid (5-CQA) was identified as the main bioactive compound responsible for the immunomodulatory effect observed by authors (Artusa et al.).

Mu et al. reported that luteolin reduced UVB-induced erythema and wrinkle formation and prevented cell viability induced by UVB. In addition, *in vitro* and *in vivo* studies showed that luteolin reduced oxidative stress, decreased activation of matrix metalloproteinases (MMPs) and increased collagen expression. Luteolin protects skin cells against UVB radiation-induced ageing *via* the SIRT3/ROS/mitogen-activated protein kinases (MAPK) axis.

In another paper, hexane, dichloromethane (DCM), and ethanol extracts of *Cannabis sativa* L. leaves presented hepatoprotective properties by preventing the depletion of antioxidant defense system (reduced glutathione, superoxide dismutase, catalase and ENTPDase) (Erukainure et al*.* ). Interactions between CBD and Δ-9-THC with the β2 adrenergic receptor of the adrenergic system was elucidated by molecular docking studies. In this way *Cannabis sativa* L. protects against oxidative-mediated hepatic injury (Erukainure et al.). In another study Jiang et al. elucidated the mechanism of action of *Oplopanax elatus* Nakai in protecting the hepatic tissue. *Oplopanax elatus* Nakai prevented an increase in alanine aminotransferase, and aspartate aminotransferase activities, affected the metabolism of APAP, and inhibited the increase in concentrations of APAP, APAP-CYS and APAP-NAC by hindering CYP2E1 and CYP3A11 activities. The protective effect of *Zornia diphylla* (L.) Pers. against carbon tetrachloride induced acute liver injury was reported by Xie et al. Zornia diphylla (L.) Pers (ZDP) decreased liver index, serum liver function indices and lipid peroxidation indices; while restoring antioxidant functions characterized by an increase in liver antioxidants (SOD, CAT and GSH) and improving the expression of inflammatory cytokines (Akt, p-Akt, NF-κB p65, IκB-α, IL-1β, IL-6 and TNF-α).

Yin et al. reported the potential antioxidant mechanism of Huolisu Oral Liquid (HLS) in serum. HLS shows antioxidant function by reducing inflammation, the release of pro-inflammatory cytokines, and mitochondrial autophagy regulation. Smeriglio et al. evaluated betalain-rich extracts against intestinal inflammation. Betanin, indicaxanthin, and prickly pear extracts were evaluated for antioxidant and anti-inflammatory activities. Prickly pear extracts possessed antioxidant and anti-inflammatory activity by inhibiting the release of reactive oxygen species (ROS) and inflammatory markers (IL-6, IL-8, and NO). Amentoflavone (AMF) alleviated inflammatory effusion and pathological injury as well as protected against Car-induced pleurisy and lung injury (Hou et al*.* ). AMF also decreased SOD and GSH depletion and increased MDA and MPO generation in mice lung tissue. AMF activated Nrf2 through keap-1 dissociation and subsequently increased heme oxygenase-1 (HO-1), NAD(P)H-quinone oxidoreductase 1 (NQO1), and γ-glutamylcysteine ligase (GCL) levels. Furthermore, AMF suppressed IL-1β and TNF-α levels and increased IL-10 levels in pleural exudate by blocking the proinflammatory NF-κB, signal transducer and activator of transcription 3 (STAT3) and extracellular signal-regulated kinase (ERK) pathways induced by Car (Hou et al*.* )

Two reviews were published in this Research Topic. In the first, Ochieng et al. highlighted the use of *Syzygium jambos* (L.) Alston in traditional medicine, its chemical composition and biological effects focusing on the antioxidant and anti-inflammatory activities. The exacerbation of oxidative and inflammatory reactions in atherosclerotic cardiovascular diseases leads to morbidity and mortality worldwide. In the review, Cao et al. highlighted the importance of antioxidant and anti-inflammatory effects of berberine in fighting atherosclerosis in experimental and clinical settings. They comprehensively discussed the interaction between berberine and intestinal microbiota, providing novel insights into the berberine usage in the management of the atherosclerotic disease. These findings contribute to the identification of novel therapeutic potentials from medicinal plants and natural products in the prevention and treatment of atherosclerotic cardiovascular diseases.

## Conclusion

Oxidative and inflammatory reactions are required processes involved in the defense and normal physiological functioning of living systems. Both Oxidative and inflammatory reactions are stimulated by/or are by-products of the salubrious physiology responsible for the host defense and neuronal transduction. However, excesses of these response systems have been associated with degenerative processes and metabolic diseases. This Editorial is a summary of salient results of articles published in the Research Topic. We here summarized the response mechanisms of the system stimulated by natural products to restore normal physiology. Medicinal plants extracts and their bioactive compounds exhibited therapeutic potentials for the management of pathological conditions related to oxidative and inflammatory processes which may play a role in reducing synthetic drug use for the treatment these complications. This Research Topic highlights the importance of natural products as modulators of oxidative stress and inflammatory processes in humans, and the interest in validation of the efficacy of plants traditionally used as remedies to fight inflammatory conditions. However, limitation is the lack of clinical studies, thus underlining that efficacy in humans is unfortunately unclear. Thus, this Research Topic demands clear efforts in clinical validation of the pharmacological activities of natural products herein described.

